# Hypoxia induces robust ATP release from erythrocytes in ApoE-LDLR double-deficient mice

**DOI:** 10.3389/fphys.2024.1497346

**Published:** 2024-11-29

**Authors:** Fatih Celal Alcicek, Jakub Dybas, Katarzyna Bulat, Tasnim Mohaissen, Ewa Szczesny-Malysiak, Magdalena Franczyk-Zarow, Katarzyna M. Marzec

**Affiliations:** ^1^ Institute for Cardiovascular Physiology, Goethe University, Frankfurt, Germany; ^2^ Jagiellonian Centre for Experimental Therapeutics, Jagiellonian University, Krakow, Poland; ^3^ Faculty of Physics and Applied Computer Science, AGH University of Science and Technology, Krakow, Poland; ^4^ Department of Biomedical Sciences, Faculty of Health and Medical Sciences, University of Copenhagen, Copenhagen, Denmark; ^5^ Department of Human Nutrition and Dietetics, Faculty of Food Technology, University of Agriculture, Krakow, Poland

**Keywords:** red blood cells, hemoglobin, ATP release, hypoxia, atherosclerosis

## Abstract

Red blood cells (RBCs) play a role in the regulation of vascular tone via release of adenosine triphosphate (ATP) into the vasculature in response to various stimuli. Interestingly, ApoE/LDLR double-deficient (ApoE/LDLR^−/−^) mice, a murine model of atherosclerosis, display a higher exercise capacity compared to the age-matched controls. However, it is not known whether increased exercise capacity in ApoE/LDLR^−/−^ mice is linked to the altered ATP release from RBCs. In this work, we characterized the ATP release feature of RBCs from ApoE/LDLR^−/−^ mice by exposing them to various stimuli *in vitro*. The results are linked to the previously reported mechanical and biochemical alterations in RBCs. 3V-induced ATP release from RBCs was at comparable levels for all groups, which indicated that the activity of adenylyl cyclase and the components of upstream signal-transduction pathway were intact. Moreover, hypoxia- and low pH-induced ATP release from RBCs was higher in ApoE/LDLR^−/−^ mice compared to their age-matched controls, a potential contributing factor and a finding in line with the higher exercise capacity. Taken together, augmented hypoxia-induced ATP release from RBCs in ApoE/LDLR^−/−^ mice indicates a possible deterioration in the ATP release pathway. This supports our previous reports on the role of the protein structure alterations of RBC cytosol in hypoxia-induced ATP release from RBCs in ApoE/LDLR^−/−^ mice. Thus, we emphasize that the presented herein results are the first step to future pharmacological modification of pathologically impaired microcirculation.

## 1 Introduction

Red blood cells (RBCs) play a prominent role in the microcirculation regulating the blood flow and consequently oxygen delivery to tissues via release of adenosine triphosphate (ATP) molecules into the vasculature in response to various stimuli (Ellsworth et al., 1995; Sprague et al., 2007; Ellsworth and Sprague, 2012). ATP is present in RBCs in millimolar concentrations and ATP molecules are released from RBCs spontaneously with a constant rate when no perturbation occurs (Montalbetti et al., 2011). Robust ATP release from RBCs can be induced by applying various physiological and pharmacological stimuli including deformation (Sprague et al., 1996; Sprung et al., 2002), low pH (Ellsworth et al., 1995), hypoxia (Bergfeld and Forrester, 1992; Sprague et al., 2010; Ellsworth et al., 2016), prostacyclin (PGI2) analogs (Sprague et al., 2010; Sridharan et al., 2012), and β-adrenergic receptor agonists (Adderley et al., 2009). The proposed pathways of ATP release from RBCs (Ellsworth and Sprague, 2012) include G proteins, adenylyl cyclase (AC), cyclic adenosine monophosphate (cAMP), protein kinase A (PKA), phosphodiesterase 3 (PDE3), cystic fibrosis transmembrane conductance regulator (CFTR), and anion channels, e.g., hemichannel Pannexin-1 (PANX1) (Sridharan et al., 2010) and voltage-dependent anion channel (VDAC) (Sridharan et al., 2012; Marginedas-Freixa et al., 2018). PANX1 (Locovei et al., 2006; Qiu and Dahl, 2009) and VDAC (Sridharan et al., 2012) proteins are expressed on RBC membranes and shown as the final conduits of ATP export from RBCs in response to various stimuli (Sridharan et al., 2010; 2012; Montalbetti et al., 2011; Leal Denis et al., 2013; Marginedas-Freixa et al., 2018). Previous studies have shown that the release of ATP from RBCs is impaired in diseases such as type 2 diabetes mellitus (Sprague et al., 2010), pulmonary hypertension (Sprague et al., 2001b), cystic fibrosis (Sprague et al., 1998), and Alzheimer´s disease (Carelli-Alinovi et al., 2016). Depletion of intracellular ATP level in RBCs was previously reported in advanced atherosclerosis (Ekstrand et al., 2017). Nevertheless, to the best of our knowledge, no study was performed to characterize the altered ATP release from RBCs in any atherosclerosis model.

ApoE/LDLR double-knockout (ApoE/LDLR^−/−^) mice model shows impaired lipoprotein clearance and is a reliable animal model to investigate the atherosclerosis disease (Lo Sasso et al., 2016; Wojewoda et al., 2016; Bar et al., 2019; Dybas et al., 2020). From an early age, hypercholesterolemia develops, and atherosclerotic lesions begin to occur between the ages of 12–16 weeks, even when fed a normal diet (Wojewoda et al., 2016; Bar et al., 2019). We have previously analyzed the various mechanical, functional, and biochemical features of RBCs isolated from ApoE/LDLR^−/−^ mice (Dybas et al., 2020; Alcicek et al., 2022; Alcicek et al., 2023). Interestingly, it was previously reported that ApoE/LDLR^−/−^ mice display unexpectedly higher exercise capacity as compared to their age-matched controls (Wojewoda et al., 2016). Despite a direct link between increased exercise capacity in ApoE/LDLR^−/−^ mice and ATP release from RBCs was not found, such dependency cannot be excluded. The lipid profile of the RBC membrane (Dybas et al., 2020) and the secondary structure of hemoglobin (Hb) in RBCs (Alcicek et al., 2023) were found to be altered in ApoE/LDLR^−/−^ mice. Both could affect the ATP release mechanism of RBCs since the lipid composition can alter the activity of channels on the membrane (Lee, 2004) such as PANX1 and VDAC and the involvement of Hb in the hypoxia-induced ATP release from RBCs was emphasized (Jagger et al., 2001; Clapp et al., 2013). Accordingly, we hypothesized that hypoxia-induced ATP release from RBCs in ApoE/LDLR^−/−^ mice is not impaired and may even by increased and could contribute to the mechanism that resulted in the reported higher exercise capacity in individuals or experimental animals with atherosclerosis. In this work, the robust ATP release from RBCs in response to various stimuli in a mouse model of atherosclerosis was comprehensively characterized for the first time.

## 2 Materials and methods

### 2.1 Animal models

Murine model of atherosclerosis (8- and 24-week-old, female, N = 4–5), ApoE/LDLR^−/−^ (Ishibashi et al., 1994), bred at University of Agriculture in Krakow, and an appropriate healthy control (8- and 24-week-old, female, N = 4–5), C57BL/6J (Aged C57BL/6J Mice - JAX, 2024), bred at the Jackson Lab (Bar Harbor, Maine, United States), were used for the experiments. Mice were housed at the animal facility of Jagiellonian Centre of Experimental Therapeutics, Jagiellonian University in Krakow, in the conditions of 12-hour light/dark cycle, standard rodent chow diet, and unlimited access to drinking water. All experiments were performed with respect to the ARRIVE guidelines and the Guidelines for Animal Care and Treatment of the European Union and authorized by the First Local Ethical Committee on Animal Testing at the Jagiellonian University in Krakow. According to the Directive 2010/63/EU (Chapter I, Article 3, Clause 1) of the European Parliament and of the Council, no additional approval is necessary when the animals sacrificed for tissue and blood collection are not subjected to any procedures beforehand. The number of mice in each given experimental group is stated in the captions of the corresponding figures.

### 2.2 Blood collection, RBC isolation, and complete blood count

Mice were euthanized via intraperitoneal injection of overdose of ketamine (100 mg/kg) and xylazine (10 mg/kg) mixture. Whole blood samples were collected directly from the right ventricle using 21G needle and gently aspirated to a 2 mL syringe containing heparin as an anticoagulant (10 units/mL). Whole blood samples were subjected to triple centrifugation (acceleration: 500 ×*g*, run time: 10 min, 4°C, soft stop) up to 1 h after collection. The plasma and buffy coat were aspirated after each centrifugation and RBCs were washed with the daily buffer solution (DBS). The DBS was prepared on the same day for each experiment, and it contained: 21 mM Tris Base, 140.5 mM NaCl, 4.7 mM KCl, 2 mM CaCl_2_, 1.2 mM MgSO_4_, 5.5 mM glucose and 76 µM bovine serum albumin, with a final pH adjusted to 7.40. All chemicals were dissolved in distilled water and the solution was filtered through a pleated filter with 0.22 µm pore size.

Immediately after collection whole blood samples were subjected to complete blood count analysis (red blood cell count (RBC), hematocrit (HCT), HGB, MCV, MCH, and MCHC) using the AbcVet hematology analyser (Horiba Medical, Montpellier, France). Additionally, complete blood count (CBC) was applied to determine the number of RBCs in each sample following the isolation.

### 2.3 Western blot analysis of PANX1 and VDAC1 proteins expression on RBC membranes

The RBC membranes were prepared by overnight freezing of isolated RBCs suspended in 0.9% NaCl (hematocrit = 10%) as described previously (Dybas et al., 2020). The mechanical damage with growing ice crystals ruptured RBC membranes, leading to the release of Hb. Subsequently, samples were thawed and centrifuged (acceleration: 3,000 ×g, 10 min, 4°C, soft braking), rinsed three times with saline and the RBC membranes were aspirated from the layer above the Hb.

The acquired isolated RBC membranes were resuspended in 100 μL of RIPA buffer supplemented with protease inhibitor and phosphatase inhibitor cocktails. Total protein amounts were measured in each sample using the BCA Protein Assay Kit (Thermo Scientific, cat. No. 23225) according to the supplier’s protocol. Then samples (the volume contains 30 μg of proteins) were mixed with Protein Loading Buffer, boiled at 95°C for 5 min, put in the ice for 2 min, and were run in an SDS-polyacrylamide gel (10%, 1.5 mm thickness) followed by transfer to nitrocellulose membrane. Blots were blocked in LiCor Intercept blocking buffer and incubated overnight with primary antibodies (anti-PANX1 (1:1000) (Proteintech Group, cat. No. 12595-1-AP) and anti-VDAC1 (1:1000) (Proteintech Group, cat. No. 55259-1-AP); each sample also has anti-beta actin (1:1000) as a loading control (Abcam, cat. No. ab8226)) separately at 4°C, followed by fluorescently tagged secondary antibodies (1:5000) (Abcam, Goat Anti-Rabbit IgG, cat. No. ab97051, and Goat Anti-Mouse IgG, cat. No. ab205719) for 1 h at RT. Blots were imaged via fluorescence using a LiCoR Odyssey CLx imaging system.

### 2.4 Determination of induced ATP release from RBCs in presence and absence of proper channel inhibitors

ATP levels were measured using ATPlite 1step luminescence ATP detection assay system (PerkinElmer, Massachusetts, United States), which is based on luciferin-luciferase technique (Strehler, 1968), according to the supplier’s protocol. Light emitted from the reaction of D-luciferin with ATP was measured in each well using a Luminometer LB9508 (Berthold Technologies, Germany). To determine ATP levels, the peak light emitted was compared with an ATP calibration curve generated during the experiment. Additionally, to make sure that only ATP was measured, isolated RBCs in all experiments were incubated with 2 UN/mL of apyrase enzyme from potatoes (Sigma Aldrich, Darmstadt, Germany), which hydrolyzes ATP.

RBC suspensions were prepared with 2.5% hematocrit to determine the release of ATP from murine RBCs in response to various stimulators in absence and presence of channel inhibitors. All defined concentrations below are the final concentrations in each sample. All incubations were performed in a heating block incubator with gentle shaking at 300 rpm.

To induce ATP release through VDAC, RBC suspensions were mixed with 1 μM iloprost (Cayman, Michigan, United States), and incubated at 37°C for 30 min. For inhibition of VDAC, isolated RBCs were incubated with 10 μM Bcl-xL BH44-23 (BCL) (Sigma Aldrich, Darmstadt, Germany) at 37°C for 25 min prior to the incubation with 1 μM iloprost.

3V cocktail (10 μM isoproterenol, 30 μM forskolin, and 100 μM papaverine) (Sigma Aldrich, Darmstadt, Germany) were mixed with RBC suspensions, and incubated at 37°C for 10 min to induce ATP release through PANX1 channel. For inhibition of PANX1, isolated RBCs were incubated with 10 μM carbenoxolone disodium salt (CBX) (Sigma Aldrich, Darmstadt, Germany) at 37°C for 5 min prior to the incubation with 3V.

To generate hypoxic conditions, 2 mL of DBS were placed into each well of 6-well plate that were seated into the gas treatment chamber NOX-E.5-GTC/TGC (Noxygen Science Transfer & Diagnostics GmbH, Elzach, Germany) set at 37°C and connected to a constant nitrogen flow (Locovei et al., 2006). Subsequently, isolated RBCs were injected to the wells with hypoxic DBS and incubated for 5 min. For inhibition of PANX1, isolated RBCs were incubated with 10 μM CBX at 37°C for 5 min prior to the injection.

A volume of prepared DBS was separated and adjusted to pH 7.03 ± 0.02. Isolated RBCs incubated in DBS with pH 7.03 ± 0.02 at 37°C for 5 min.

At the end of every incubation, all samples were subjected to centrifugation (acceleration: 1,000 ×g (Montalbetti et al., 2011), run time: 2 min, 4°C, soft stop) to obtain the supernatant for ATP determination. Two technical replicates, 50 μL of supernatant from each sample, were taken for the measurement. Values were normalized to picomoles per 4 × 10^8^ RBCs. Difference in the ATP levels were defined by analysis of the ratio of ATP levels after incubation to those before incubation, called the basal extracellular ATP. While the absolute ATP levels in RBCs from these groups have been previously reported (Alcicek et al., 2022), in the present study, we used ratios to emphasize the relative impact of ATP stimulators on RBCs. This approach facilitates clearer comparisons of induced ATP release, which did not exhibit a similar trend to intracellular or basal extracellular ATP levels across the studied groups.

### 2.5 Evaluation of hemolysis

Presence of Hb in the supernatant was determined with use of a double beam spectrophotometer Lambda 950 (Perkin Elmer, Massachusetts, United States) and evaluated to exclude possible significant hemolysis, not more than 1%, occurred during the incubation of isolated RBC suspensions at 37°C that may affect the determined extracellular ATP levels of RBCs. Absorption spectra were recorded in the 300–700 nm spectral range using a 10 mm path length cuvette. The level of hemolysis was assessed based on the absorbance at 415 nm (Kampen and Zijlstra, 1966). The supernatant from each sample was diluted 50 times with 0.9% NaCl solution prior to measurement.

### 2.6 Statistical analysis of data

All data were analyzed in OriginPro 2018 (OriginLab, Massachusetts, United States) software. Statistics were calculated according to the results of normality assessment performed with using of Shapiro-Wilk test. If the data met normality requirement, significance was checked with one-way ANOVA model with Fishers Least Significant Difference (LSD) test and results were presented as bar diagrams with mean and standard error (SE). Otherwise, Kruskal–Wallis ANOVA nonparametric test was performed, and results were shown as box diagrams containing mean, median, and interquartile range together with min-max whiskers.

## 3 Results

### 3.1 3V-induced ATP release from RBCs in the presence and absence of CBX

**FIGURE 1:**
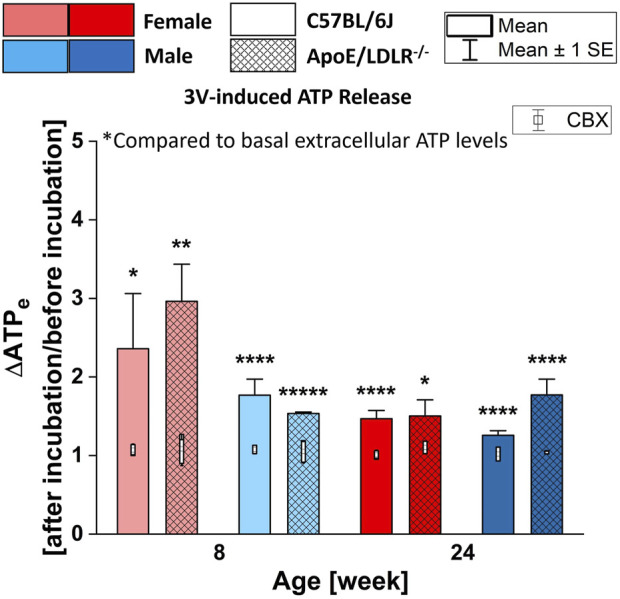
Comparison of the released ATP levels from RBCs isolated from female and male, 8- and 24-week-old, C57BL/6J and ApoE/LDLR^−/−^ mice (N = 3–5) in response to 3V cocktail (contains 10 μM isoproterenol, 30 μM forskolin, and 100 μM papaverine) in absence and presence of 10 μM CBX. Differences in the ATP levels were defined by the ratio of ATP levels at after incubation to before incubation.

Stimulation with 3V cocktail (10 μM isoproterenol, 30 μM forskolin, and 100 μM papaverine) resulted in robust and statistically significant release of ATP in all studied samples compared to their basal extracellular ATP levels (Figure 1). However, no significant disease-, sex-, and age-related differences were observed among the studied groups. Additionally, RBCs incubated with 10 μM CBX (carbenoxolone, Pannexin1 inhibitor) did not release ATP in response to 3V cocktail in any of studied mice group.

### 3.2 Hypoxia-induced ATP release from RBCs in the presence and absence of CBX

**FIGURE 2:**
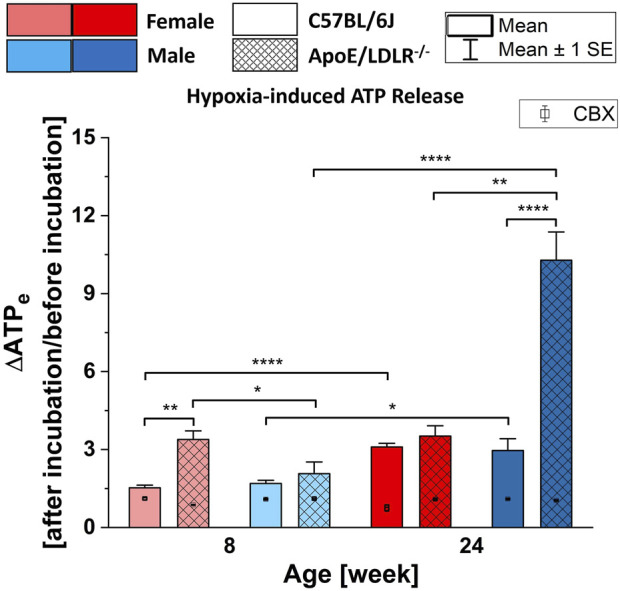
Comparison of the released ATP levels from RBCs isolated from female and male, 8- and 24-week-old, C57BL/6J and ApoE/LDLR−/− mice (N = 3–6) in response to hypoxia in absence and presence of 10 µM CBX. Differences in the ATP levels were defined by the ratio of ATP levels at after incubation to before incubation. Significances are compared to the basal extracellular ATP levels.

Robust release of ATP from RBCs in all studied samples in response to hypoxia resulted in significant differences in each studied group (Figure 2). Moreover, significant disease-, sex-, and age-related differences were observed in the levels of hypoxia-induced ATP release from RBCs.

We observed higher release of ATP from RBCs isolated from ApoE/LDLR−/− mice compared to their age-matched controls when RBCs were subjected to hypoxic environment, which was significant between 8-week-old females and 24-week-old males. RBCs acquired from 8-week-old female ApoE/LDLR−/− mice released significantly higher ATP in response to hypoxia compared to age-matched males while RBCs acquired from 24-week-old male ApoE/LDLR−/− mice released significantly higher ATP compared to age-matched females. No sex-related difference was observed in both 8- and 24-week-old control mice. Interestingly, hypoxia-induced ATP release from RBCs isolated from female and male control mice, as well as male ApoE/LDLR−/− mice, were increased with age. However, no age-related differences were observed in female ApoE/LDLR−/− mice. Additionally, when isolated RBCs were incubated with 10 μM CBX prior to be subjected to hypoxia, released ATP levels showed significant decrease in all studied groups.

### 3.3 Low pH-induced ATP release from RBCs

**FIGURE 3:**
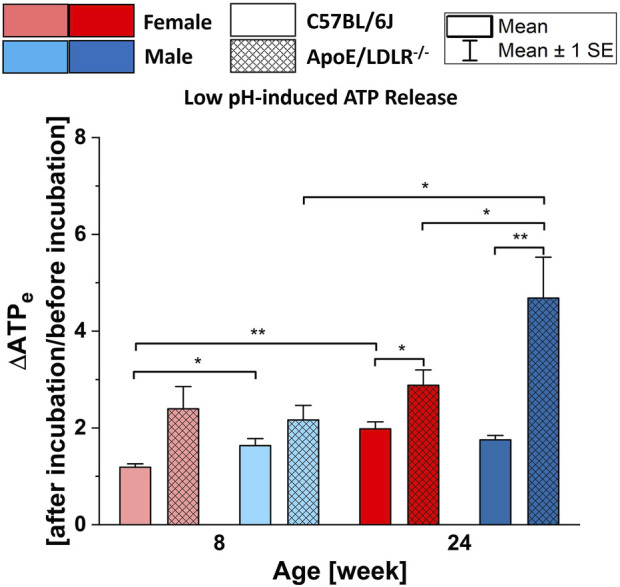
Comparison of the released ATP levels from RBCs isolated from female and male, 8- and 24-week-old, C57BL/6J and ApoE/LDLR−/− mice (N = 3–8) in response to low pH (7.03 ± 0.02) environment. Differences in the ATP levels were defined by the ratio of ATP levels at after incubation to before incubation.

In all studied samples, RBCs released significantly higher ATP in response to low pH compared to their basal extracellular ATP levels (Figure 3), except for 8-week-old female C57BL/6J mice which showed no significance. Moreover, significant disease-, sex-, and age-related differences were observed in the levels of low pH-induced ATP release from RBCs.

Low pH-induced ATP release from RBCs was significantly higher in 24-week-old female and male ApoE/LDLR−/− mice compared to age-matched controls. RBCs acquired from 8-week-old female and male ApoE/LDLR−/− mice also released higher levels of ATP but the difference was not significant compared to age-matched controls. We observed significantly higher low pH-induced ATP release from RBCs in 8-week-old male control mice, as well as 24-week-old male ApoE/LDLR−/− mice, compared to age-matched females. However, no sex-related difference was observed in 8-week-old ApoE/LDLR−/− mice and 24-week-old control mice. Low pH-induced ATP release from RBCs significantly increased with age in female control and male ApoE/LDLR−/− mice while no age-related difference was observed in male control and female ApoE/LDLR−/− mice.

## 4 Discussion

ATP release from RBCs in response to different stimuli is regulated by distinct signaling pathways. Stimulation of β-adrenergic or prostacyclin receptors activates the heterotrimeric stimulatory G protein (G_s_), whereas subjecting the RBCs to deformation or hypoxia results in the activation of the heterotrimeric inhibitory G protein (G_i_) (Olearczyk et al., 2001; Olearczyk et al., 2004b; Olearczyk et al., 2004a). Moreover, these signaling pathways include adenylyl cyclase, cyclic AMP (cAMP), protein kinase A (PKA), and CFTR (Ellsworth and Sprague, 2012). Briefly, cAMP is synthesized from ATP by adenylyl cyclase upon stimulation by either G protein. PKA is activated by elevated cAMP levels and, subsequently, CFTR; eventually resulting in ATP release. These components of the signaling pathways for ATP release from RBCs are well characterized. It appears that the initiating stimulus determines the final ATP conduit. For instance, VDAC is the final conduit for ATP release from RBCs in response to PGI_2_ analogs (Sridharan et al., 2012), while hypoxia-induced release of ATP from RBCs occurs through PANX1 (Sridharan et al., 2010).

Although in various diseases this mechanism is impaired (Sprague et al., 1998; Sprague et al., 2001b; Sprague et al., 2010), the effect of atherosclerosis on the ATP release from RBCs has never been studied. Intracellular ATP levels in RBCs from the studied groups have been previously published (Alcicek et al., 2022), revealing differences based on age and sex in ApoE/LDLR−/− mice. The current data show that the variations in ATP release observed in this study do not directly correlate with intracellular ATP levels, suggesting that other factors than just intracellular ATP levels have an impact on the differences in ATP release. To the best of our knowledge, ATP release feature of RBCs in mice model of atherosclerosis is comprehensively characterized for the first time in ApoE/LDLR^−/−^ mice. The findings are linked to the previously reported mechanical and biochemical alterations of RBCs.

### 4.1 3V-induced ATP release from murine RBCs

β-adrenergic receptor agonists induce robust release of ATP from RBCs (Sprague et al., 2007; Ellsworth and Sprague, 2012). Briefly, stimulation of β-adrenergic receptors induces G_s_ protein, results in the activation of adenylyl cyclase, and increases the cAMP (Ellsworth and Sprague, 2012). Direct activation of adenylyl cyclase also induces ATP release from RBCS via the increase in cAMP (Sprague et al., 2001a). 3V cocktail is a very efficient combination in order to enhance a cAMP-dependent release of ATP from RBCs (Montalbetti et al., 2011; Marginedas-Freixa et al., 2018).

3V cocktail contains isoproterenol, a potent nonselective β-adrenergic receptor agonist, forskolin, an adenylyl cyclase activator, and papaverine, a phosphodiesterase inhibitor; all cause the cAMP increase in RBCs which leads to the release of ATP through PANX1 (Sprague et al., 2001a; Adderley et al., 2010; Montalbetti et al., 2011; Leal Denis et al., 2013; Alvarez et al., 2014; Marginedas-Freixa et al., 2018). In this work, 3V-induced ATP release from murine RBCs was determined in absence and presence of the PANX1 channel inhibitor CBX. Our findings indicate that the 3V cocktail induced a robust release of ATP from RBCs in all studied samples (Figure 2). Although significant amount of ATP is released from RBCs in response to 3V cocktail exposure in all studied groups, no significant disease-, sex-, and age-related differences were determined among these groups. Furthermore, ATP release in response to the 3V cocktail is inhibited by CBX in all studied groups.

3V cocktail induces the accumulation of cAMP in RBCs by both direct and indirect activation of adenylyl cyclase and by the inhibition of phosphodiesterase. Therefore, our data are consistent with the hypothesis that cAMP-dependent release of ATP from RBCs through PANX1 conduit is intact for all groups. However, it is not certain if there are impairment other steps in the transduction signaling pathways. For instance, G_s_ protein-related activation of adenylyl cyclase, or even its direct activation by forskolin, could be impaired and the effect of papaverine may solely result in the cAMP accumulation. Or adenylyl cyclase was activated directly by forskolin but not through the G_s_ protein-involved pathway. Nevertheless, further studies to investigate each component of the signaling pathway of ATP release from RBCs are needed to thoroughly understand the ATP release mechanism of RBCs in this model of atherosclerosis.

### 4.2 Hypoxia-induced ATP release from murine RBCs

Hypoxia is one of the first ever reported inducers of ATP release from RBCs (Bergfeld and Forrester, 1992). There have been a number of studies with various species that investigated hypoxia-induced ATP release from RBCs (Ellsworth et al., 1995; Dietrich et al., 2000; Olearczyk et al., 2004b; Sprague et al., 2009; Sprague et al., 2010) and uncovered the responsible components in its signal-transduction pathway (Sprague et al., 2007; Sridharan et al., 2010). Briefly, hypoxia stimulates the heterotrimeric G protein G_i_ on RBC membranes that results in the activation of adenylyl cyclase, accumulation of cAMP, involvement of PKA and CFTR, and release of ATP through PANX1 conduit, respectively. The same components are included in the signaling pathway defined in 3V-induced ATP release, except for the G_i_ protein. Ordinarily, G_i_ proteins inhibit adenylyl cyclase activity, however, it has been shown that the βγ-subunits of G_i_ can activate some adenylyl cyclase subtypes, which are present in RBCs (Sprague et al., 2005). Alterations in the expression of G_i_ protein on RBC membranes could affect the released ATP levels in response to hypoxia. For instance, reduced expression of G_i_ is associated with the impaired ATP release from RBCs of humans with T2DM (Sprague et al., 2006). Moreover, alterations in the expressions of both G_s_ and G_i_ proteins on RBC membranes of humans with atherosclerosis were reported (Kots et al., 1993); yet, to the best of our knowledge, no study was performed to investigate the hypoxia-induced ATP release from RBCs in any model of atherosclerosis.

Interestingly, ApoE/LDLR^−/−^ mice exhibit higher exercise capacity compared to their age-matched controls (Wojewoda et al., 2016). However, it is not known whether increased exercise capacity in ApoE/LDLR^−/−^ mice is linked to altered ATP release from RBCs, hypoxia-induced in particular. Following an exercise, the plasma levels of nucleotides including ATP were increased (Yegutkin et al., 2007); and the importance of plasma ATP levels during the exercise has been studied (Farias et al., 2005). Although ATP release from RBCs was shown to be impaired in various diseases (Sprague et al., 1998; Sprague et al., 2001b; Sprague et al., 2010), intriguingly, iloprost-induced ATP release from RBCs isolated from humans with T2DM is greater than in those from healthy humans (Sprague et al., 2010). We investigated the effect of iloprost on RBCs isolated from ApoE/LDLR−/− mice as well, however, our findings indicate that therapeutic concentrations of iloprost did not cause a robust ATP release from RBCs in any studied group (Supplementary Figure S2). Moreover, an increase in the expression of G_i_ protein, which initiates the signal-transduction pathway of hypoxia-induced ATP release, on RBC membranes acquired from humans with atherosclerosis was reported (Kots et al., 1993). Based on the previous results and results presented herein, we hypothesized that hypoxia-induced ATP release from RBCs in ApoE/LDLR^−/−^ mice is not impaired but enhanced; and consequently, could contribute to the mechanism that resulted in the reported higher exercise capacity. Accordingly, hypoxia-induced ATP release from RBCs in ApoE/LDLR^−/−^ mice compared to age-matched controls was investigated and related to the duration of disease, sex, and age-dependent differences for the first time. In addition, CBX was applied to show the involvement of PANX1 channels in the hypoxia-induced ATP release from murine RBCs in this model.

The results of 3V-induced ATP release from RBCs experiments suggested that in our model of atherosclerosis the signal-transduction pathway is intact from the adenylyl cyclase activation step which leads to the release of ATP through PANX1 conduit, but that the activation of β-adrenergic receptors could be impaired due to either lowered expression or loss of sensitivity and/or the decreased G_s_ protein expression. Our findings indicate that RBCs isolated from ApoE/LDLR^−/−^ mice released higher amounts of ATP in response to hypoxia, compared to their age-matched controls, what could be linked to an increase in the expression of G_i_ protein on RBC membranes, which was reported in RBCs of humans with atherosclerosis (Kots et al., 1993). The decreased G_i_ protein and increased G_s_ protein expressions on the RBCs acquired from humans with T2DM were strongly associated with the impairment of hypoxia-induced ATP release and greater iloprost-induced ATP release, respectively (Sprague et al., 2010). We may suggest that such an increase in the G_i_ protein expression could be associated with the observed increased hypoxia-induced ATP release from RBCs in ApoE/LDLR^−/−^ mice, since the upstream components of the signaling pathway are intact. Furthermore, hypercholesterolemia-related increased activity of adenylyl cyclase may be contributed to the higher hypoxia-induced ATP release from RBCs in ApoE/LDLR^−/−^ mice as well.

More importantly, the involvement of hemoglobin in the hypoxia-induced ATP release from RBCs was emphasized previously (Jagger et al., 2001; Clapp et al., 2013). Hemoglobin interacts with proteins from RBC membranes, including band 3 (Rauenbuehler et al., 1982). It is shown that the deoxyhemoglobin competes with ankyrin, a cytoskeletal protein, in order to bind to band 3 on the RBC membrane (Stefanovic et al., 2013). Namely, deoxyhemoglobin-band 3 binding may result in a weakened membrane-cytoskeleton interaction and leads to G_i_ activation that initiates the ATP release signaling pathway. A conformational change in hemoglobin from oxygenated to deoxygenated state was suggested to be necessity for hypoxia-induced release of ATP from RBCs (Jagger et al., 2001). Although the transition of oxy-deoxy hemoglobin occurs simultaneously as a physiological process of tissue oxygenation, the highlighted protein alterations could be an important component in the hypoxia-induced ATP release from RBCs (Alcicek et al., 2023). Other than oxy-deoxyHb levels, monitoring methemoglobin (metHb) levels is crucial for ensuring sample consistency, as even minor variations could influence outcomes; however, in our studies, we consistently observed metHb levels below 1.5%, as previously published, which we consider negligible and unlikely to impact our experimental results. Accordingly, we hypothesize that increased hypoxia-induced ATP release from RBCs in ApoE/LDLR^−/−^ mice could be partially linked to the reported secondary structure alterations in hemoglobin in such mice model (Alcicek et al., 2023). This could affect hemoglobin-band 3 interaction-related stimulation of G_i_ activation. Consistently, the observed highest hypoxia-induced ATP release from RBCs was in 24-week-old male ApoE/LDLR^−/−^ mice where the highest alterations in the secondary structure of hemoglobin were observed as well. Moreover, the previously reported correlation between the greater iloprost-induced ATP release from RBCs isolated from humans with T2DM compared to healthy subject and HbA1c levels (Sprague et al., 2010), which is a modified hemoglobin species that exhibits secondary structure alterations (Ye et al., 2016), may support our proposed relationship between the altered Hb and hypoxia-induced ATP release from RBCs in ApoE/LDLR^−/−^ mice. Nevertheless, for the purpose of comprehensively understanding the hypoxia-induced ATP release mechanism of RBCs in mice model of atherosclerosis further studies are required.

Additionally, incubating RBCs with PANX1 channel inhibitor, CBX, prior to the exposure of hypoxia resulted in significantly reduced release of ATP from RBCs in all studied groups. Accordingly, in line with the previous studies (Sridharan et al., 2010), we provide additional support for the hypothesis that PANX1 is the main conduit for hypoxia-induced ATP release from RBCs in this mouse model of atherosclerosis. Furthermore, the expression of PANX1 and VDAC1 proteins on the RBC membranes of ApoE/LDLR−/− mice are shown for the first time (Supplementary Figure S1).

### 4.3 Low pH-induced ATP release from murine RBCs

Exposure of RBCs to low pH at a level that is in the physiological range for RBCs, i.e., 7.0–7.3 (Kaniewski et al., 1994), has been demonstrated as one of the stimuli that can initiate ATP release from RBCs (Ellsworth et al., 1995). Although it was shown that low pH induces ATP release from RBCs with a similar robustness to hypoxia exposure (Ellsworth et al., 1995), comprehensive studies regarding the low pH-induced ATP release from RBCs remained scarce.

Blood and muscle pH are decreased in humans following exercise (Hermansen and Osnes, 1972). Therefore, in addition to the lowered oxygen levels, low pH could also induce the ATP release from RBCs and may contribute to the regulation of the blood flow of the tissues during the exercise. Moreover, low pH-induced ATP release from RBCs may also participate in the reported results that higher exercise capacity of ApoE/LDLR^−/−^ mice (Wojewoda et al., 2016). Accordingly, in the present study, low pH (7.03 ± 0.02)-induced ATP release from RBCs was investigated.

We observed significantly higher ATP release from RBCs in response to low pH compared to their basal levels in all studied groups. Our findings exhibited almost identical patterns with the hypoxia-induced ATP release from RBCs among these groups. RBCs from ApoE/LDLR^−/−^ mice released higher amount of ATP in response to low pH compared to their age-matched controls. Moreover, low pH-induced ATP release from RBCs was increased with age progression. Taken together, these findings suggest that the signal-transduction pathway for low oxygen- and low pH-induced ATP release could be very much alike, maybe even identical in RBCs from ApoE/LDLR^−/−^ mice. Accordingly, it is possible that increased G_i_ protein expression on RBC membranes of humans with atherosclerosis (Kots et al., 1993) and hemoglobin alterations, could also lead to enhanced low pH-induced ATP release from RBCs in ApoE/LDLR^−/−^ mice. (Huang et al., 2013).

In conclusion, this study provides a detailed characterization of ATP release mechanisms in RBCs of ApoE/LDLR−/− mice. Notably, the 3V-induced ATP release experiments demonstrated that these RBCs possess an intact cAMP-dependent ATP release signal-transduction pathway. Results from hypoxia- and low pH-induced ATP release experiments highlight a strong correlation between altered hemoglobin structure and ATP release, further supporting the hypothesis that RBCs in this mouse model may contribute to enhanced exercise capacity. However, further research is required to fully elucidate the signaling pathways involved and their modifications in the context of atherosclerosis.

Importantly, this work holds clinical relevance, as the characterization of changes in these pathways in atherosclerosis may position RBCs as a novel therapeutic target for developing strategies to treat vascular diseases in human (Kontidou et al., 2023). Additionally, further investigation into the role of hemoglobin and other altered components of the ATP release mechanism in ApoE/LDLR−/− mice could potentially pave the way for pharmacological interventions to restore pathologically impaired microcirculation.

## Data Availability

The datasets presented in this study can be found in online repositories. The names of the repository/repositories and accession number(s) can be found below: Fatih Celal Alcicek (2023), “ATP_release_dataset”, Mendeley Data, V1, doi: 10.17632/ncmfyv7tsh.1.
