# Supplementary material for: Hypoxia induces robust ATP release from erythrocytes in ApoE-LDLR double-deficient mice
Source: Front Physiol. 2024 Nov 29;15:1497346. doi: 10.3389/fphys.2024.1497346 (PMC11638198; doi:10.3389/fphys.2024.1497346)
Supplement: Supplementary file 1 [file DataSheet1.pdf]

Supplementary Material

1 Supplementary Figures

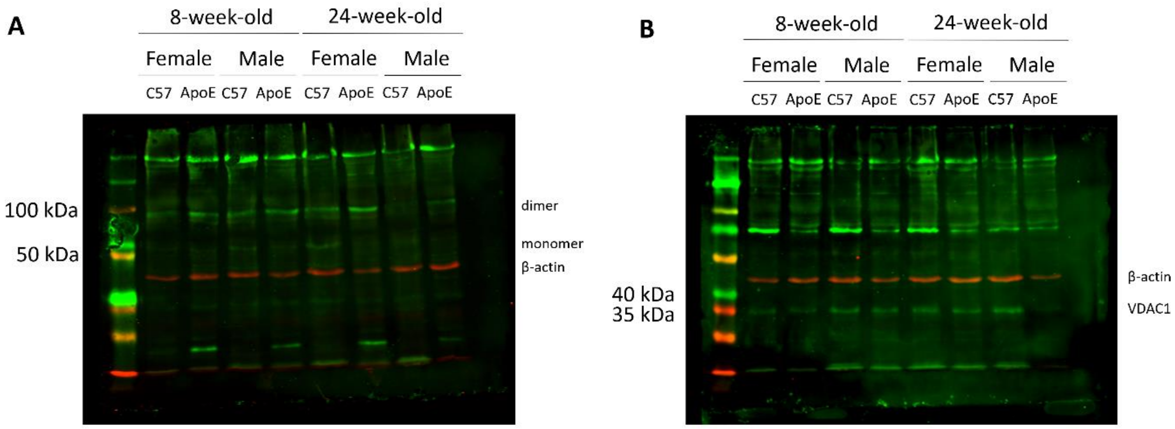

Supplementary Figure 1. Immunoblot of PANX1 and VDAC1 expression in murine RBC membranes. The original blots are displayed. Beta-actin was used as a loading control.

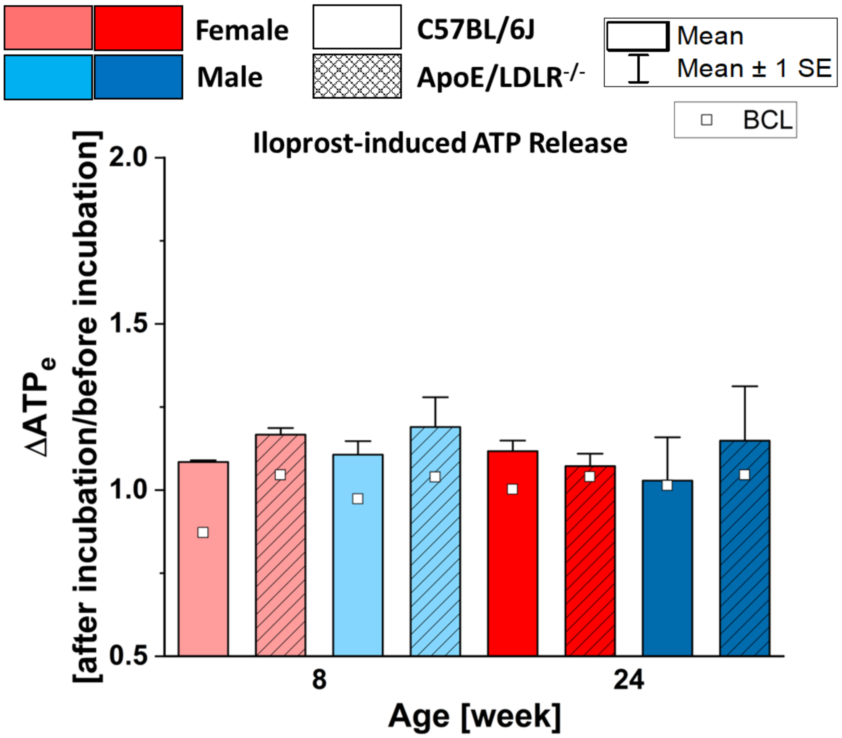

Supplementary Figure 2. Comparison of released ATP levels from RBCs isolated from female and male, 8- and 24-week-old, C57BL/6J and ApoE/LDLR-/- mice (N = 3-5) in response to 1 μM iloprost

in absence and presence of 10 μM BCL. Difference in the ATP levels were defined by the ratio of ATP levels at after incubation to before incubation.
